# The most appropriate cervical dilatation for massage to reduce labor pain and anxiety: a randomized clinical trial

**DOI:** 10.1186/s12905-022-01864-1

**Published:** 2022-07-07

**Authors:** Samira Shahbazzadegan, Roya Nikjou

**Affiliations:** Department of Midwifery, School of Nursing and Midwifery, Ardabil University of Medical Sciences, Ardabil, Iran

**Keywords:** Massage, First labor stage, Labor pain, Anxiety, Clinical trial, Randomized controlled trial (RCT)

## Abstract

**Background:**

Managing labor pain by performing massage is one of the useful strategies to reduce the rate of cesarean section and increase the tendency of women for pregnancy. Therefore, it is necessary to determine the best time for massage therapy to reduce the labor pain and anxiety. In this regard, the present study was conducted to determine the cervical dilatation appropriate for performing massage in order to reduce the labor pain and anxiety.

**Methods:**

This randomized clinical trial study was conducted on 60 nulliparous pregnant women. Eligible participants with active phase of labor were divided randomly into two groups. The intervention group received the massage three times in of dilatation 5–7–9 cm for 20 min each time by same person without the use of oil in the LDR, based on Kimber massage instructions. In the control group, all routine care was performed except massage. Pain intensity was assessed using pain ruler. Demographic and anxiety data were collected through questionnaires.

**Results:**

The difference between the mean pains in the studied groups was significant in 7 cm (p < 0.0001) of cervical dilatation but was not significant in 5 cm (p = 0.084) and 9 cm (p = 0.591) dilatation. Massage effectively decreased pain intensity. The mean maternal anxiety was not significant at the beginning of the study, but was significant after performing massage (p < 0.0001) and anxiety score in the massage group decreased from 63.36 ± 5.28 (severe anxiety) at the beginning to 42.60 ± 5.83 (moderate anxiety) at the end of the study. In the control group, it increased from 66.33 ± 7.66 to 67.1 ± 5.65.

**Conclusion:**

The appropriate dilatation of cervix for massage in order to reduce labor pain was observed in 7 cm. Also, massage had a significant effect on reducing anxiety. Therefore, massage is recommended as a routine care in 7 cm cervical dilatations.

***Trial registration*:**

This trial was registered with the Iran Trial Center (trial ID: IRCT20140118016255N5). https://en.irct.ir/trial/28120

## Background

Labor pain is one of the reasons that reduces the women’s tendency to get pregnant [1]. Labor pain is caused by stretching and enlarging the walls of the cervix, vagina and perineum, as well as severe contractions of the uterine wall [2]. Pain and anxiety affect the delivery process; severe labor pain and anxiety can increase adrenaline and cortisol, leading to decreased uterine activity and prolonged labor [3]. With the prolongation of labor, the risk of infection, physical and nervous damage, and the fetus and infant death increase, and the mothers expose to bleeding, postpartum infection, mental distress, insomnia, and fatigue [1, 4]. Childbirth pain can lead to loss of maternal psychological control during delivery, traumatic childbirth and mental disorders. On the other hand, elongation of labor pain can reduce the progression of labor and increase the need for cesarean section and labor stimulation [4]. Persistence of pain and anxiety during labor also affects the respiratory system, blood circulation, endocrine glands, and other body functions, and therefore can lead to increased dystocia [3]. Pain management is an important factor in increase of women’s tendency for fertility and natural birthing process. On the other hand, labor pain also increases cesarean section (CS) rates [1]. Cesarean section rates have been increasing globally. Iran has one of the highest CS rates in the world (47.9%). Massage helps to identify effective strategies to reduce the CS rate [5].

Unlike the popular belief “obvious, inevitable pain occurs during delivery and must be tolerated”, today the relief of labor pain has been proposed as a new approach [6]. Pleasant experience of delivery and reducing pain with massage therapy is one of the solutions [7]. Massage is a systematic touch and manipulation of the soft tissues of the body that is increasingly being used as an adjunctive therapy for stress relief and to promote relaxation and wellbeing as an alternative for pharmacologic or invasive forms of analgesia during labor [8]. Some studies have shown that massage reduces labor pain [4, 9]. Pawale and Salunkhe in 2020 reported that back massage was effective in reducing pain during the first stage of labor in primipara mothers [10]. Baljon et al. in 2022 declared that massage lowered labor pain intensity and anxiety [11]. In another study combination of pelvic rocking and back massage had a significant effect on pain and duration of labor [12]. Performing massage also accompanied by fewer requests for epidural analgesia [13].

Furthermore, performing massage to relieve labor pain and applying this knowledge is an expensive and time-consuming procedure for organizations. Today, knowledge management is a more important category than knowledge itself, and organizations seek to establish it and try to explain how to turn knowledge into skills. Therefore, due to limited resources and personnel, applying massage in most effective time, especially for a tender population like pregnant women seems to be very important [14]. Anxiety occurs in stressful situations such as childbirth that can affect the pain and labor Process [4]. Therefore, decreasing of anxiety is also important.

The literature supports the use of massage in pregnancy and labor; however, its evidences have been increased over the past 30 years [9–11]. Massage has been shown to be valuable for pregnant women [4]. Determining the most effective time for massage to control labor pain is critical for proper management of this method and there were limited researches in this field. Therefore, the present study was conducted in order to determine the appropriate dilatation for applying massage to reduce the labor pain and anxiety.

## Methods

**Fig. 1:**
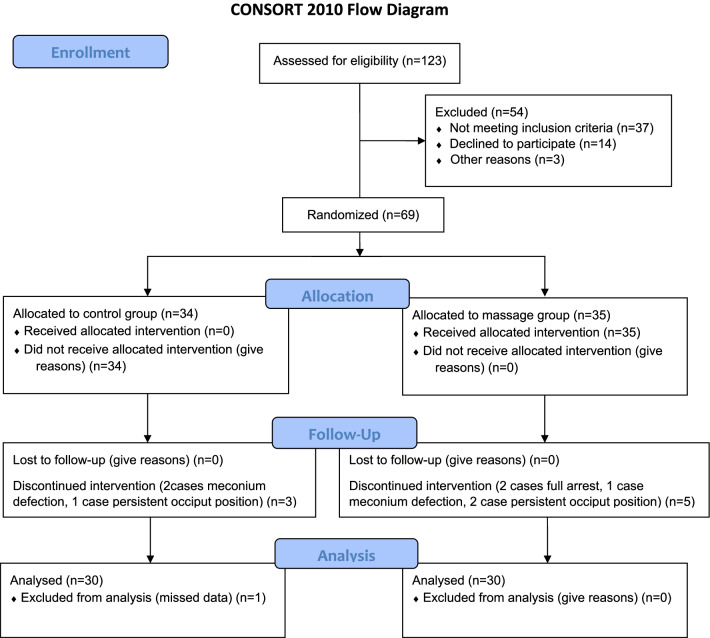
Flow diagram of recruitment and retention of participants in the study

This study conducted and reported on the basis of the Consolidated Standards of Reporting Trials (CONSORT) 2010 statement. A flow diagram of the randomized controlled protocol is shown in Fig. 1.This study registered on 15/01/2019 at https://en.irct.ir/trial/28120 adheres to CONSORT guidelines.

### Study design and sample size

This randomized, controlled clinical trial was carried out after obtaining ethics approval. The study population was 123 primiparous women who referred to Alavi Hospital in Ardabil city in an official time during 2020–2021. Ardabil is the center of Ardabil province in the northwest of Iran. Since all participants were referred to a public hospital for delivery, they were approximately in the same socioeconomically class.

Inclusion criteria included married, normal medical and family conditions, having a normal, wanted, singleton and full-term pregnancy, vertex presentation, onset cervix dilatation, absence of neurological and psychological problems, having massage criteria (no skin disease and any lesions in the massage area). Exclusion criteria were feto-maternal high-risk situations.

Sample size was calculated 30 number for each study group using G Power software whit α = 0.05, power = 0.85, effect size = 0.71 and PASS Software with α = 0.05, power = 0.96, SD = 2, effect size = 0.5.

Allocation to study groups carried out by simple randomization method. Participants were chosen, one sealed envelope out of ten (5 massage group, 5 control group), offered by the researcher. This process was continued for all participants with inclusion criteria. From 123 pregnant women, 37 persons did not have the inclusion criteria, 20 women declined to participate, 8 women discontinued intervention (2 cases with full arrest, 3 cases of meconium defection, 3 cases of persistent occiput position), and one excluded from the analysis (refer to Fig. 1 for the CONSORT flow diagram). Finally, 60 women (in two groups of massage (30 person) and control (30 person)) completed the study process (see the CONSORT flow diagram). Written informed consent was obtained from all participants. Demographic information including the age, height, pre-pregnancy weight, current weight, educational level and job was collected with a demographic questionnaire. Validity of demographic questionnaire approved by 10 expert persons. Maternal height and weight were routinely measured when entering the maternity ward. Pre-pregnancy weight was obtained from the mother's personal diary used for recording maternal care during pregnancy.

### Intervention

**Fig. 2:**
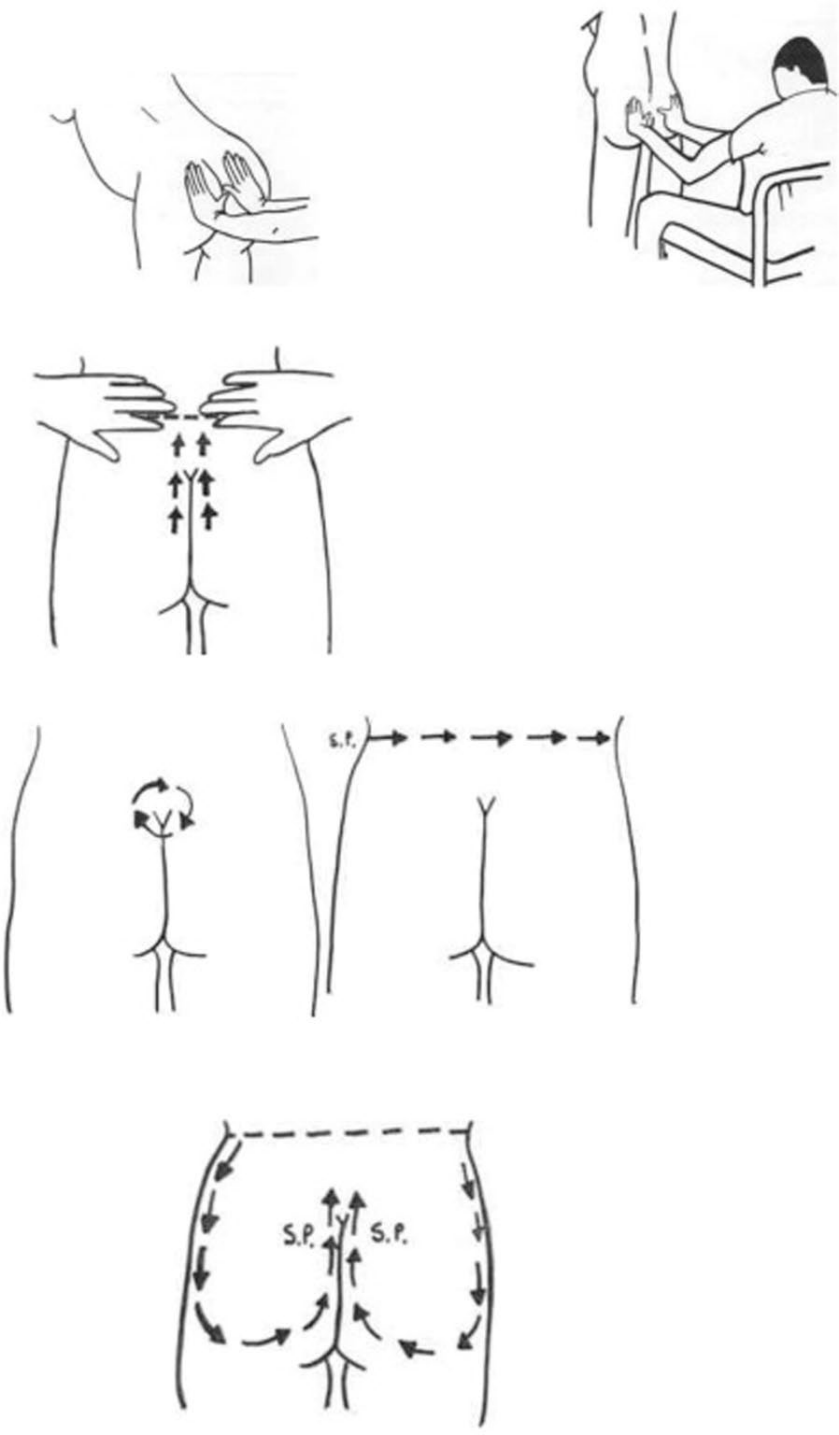
Kimber massage guideline

At the beginning of the study, in the active phase of labor, pain intensity and anxiety of participants were assessed. In the massage group from dilatation of 4 cm of the cervix to delivery, back massage was performed 3 times in dilatation of 5–7–9 cm based on Kimber massage instructions. The massage was done without the use of oil in the position desired by the mother in the LDR (Labor, Delivery, and Recovery) private room. This technique was applied between T10 and S4 according to Fig. 2 [9] for 20 min each time. In order to control the amount of received massage and the uniformity of the intervention and to increase the accuracy of the study, massage, cervix examination and measurements were performed by the same researcher. In the control group, the routine maternity ward care was performed except massage. In addition, the researcher accompanied by participants in the control group as done for the massage group.

### Measurement

The primary outcome was the change in pain intensity and anxiety. Pain intensity was marked four times in each group according to the visual standard pain ruler. The ruler used had 10 cm length, which zero at one end indicates "no pain" and number 10 represents the "greatest possible pain". The reliability and validity of the instrument were also confirmed [15, 16].

The participants' anxiety was assessed at the beginning and the end of the study with the State‐Trait Anxiety Inventory (STAI) [17]. In this questionnaire, the total numerical scores were obtained between 20 and 80 in a way that the anxiety rate of 20–31 attributed to mild anxiety, 32–42 to low-moderate anxiety, 43–53 to moderate-high, 54–64 to relatively severe, 65–75 to severe, and 76 and above to very severe anxiety. The reliability and validity of the Persian version of the STAI anxiety scale for pregnancy were studied by Mortazavi et al. [18] in a sample of Iranian women. The duration of labor was calculated from the time of entering into the active phase of labor until the time of fetal birth. The Secondary outcome was the determining appropriate cervical dilatation for massage. The appropriate cervical dilatation for massage was considered as a dilatation with highest reduction in pain intensity.

### Statistical analysis

Data were analyzed using SPSS software version 26. The categorical variables of demographic characteristics were analyzed by Chi-square and the independent t-test was used for continuous variables. The Analysis of Covariance (ANCOVA) was used to compare the means of pain and anxiety data. The p < 0.05 was considered as significant.

### Ethical considerations

After obtaining permission from the Iran National Committee for Ethics in Biomedical Research (IR.ARUMS.REC.1396.204). Study registered in the Iranian Registry of Clinical Trial (IRCT20140118016255N5) in15/01/2019, https://en.irct.ir/trial/28120. Then we conducted the study in Alavi hospital in Ardabil-Iran. Before completing the questionnaire, participants were informed about the objectives of the study, and informed consent was obtained from all participants included in the study. They were assured of the confidentiality of information.

## Results

In this study, 60 pregnant women on the threshold of delivery were studied. Demographic information of the participants included the mean height, pre-pregnancy weight and current weight, pre-pregnancy BMI and current BM, age, job and educational level in the two groups were shown in Table 1. In terms of education, 3 person (5%) of participants were illiterate, 50 (83.3%) were in primary and high school level and 7 (11.7%) were diploma and higher. Also, 5 persons (8.3%) were employed and 55 persons (91.7%) were housewives (Table 1). There was no significant difference between control and massage groups in demographic variables.

**Table 1:** Demographic informations of the participants

Variable		Control group (N = 30)	Massage group (N = 30)	*p*
Age (year)		23.19 ± 4.86	24.63 ± 4.08	0.19*
Height (cm)		158.8 ± 7.20	161.9 ± 7.51	0.54*
Pre-pregnancy weight (kg)		59.96 ± 5.06	57.10 ± 7.8	0.09*
Current weight (kg)		70.73 ± 5.60	68.46 ± 5.5	0.27*
Pre-pregnancy BMI (kg/m^2^)		21.79 ± 2.92	22.37 ± 1.31	0.33*
Current BMI (kg/m^2^)		26.15 ± 3.20	26.31 ± 1.42	0.79*
Occupation (number (%))	Housewife	27 (90%)	28 (93%)	0.64^+^
	Employee	3 (10%)	2 (6.6%)	
Education (number (%))	Illiterate	2 (6.6%)	1 (3.3%)	0.75^+^
	Primary and high school	24 (80%)	26 (86.6%)	
	Diploma and higher	4 (13.3%)	3 (10%)	

^*^Independent t-test for continuous variables, ^+^Chi-square for categorical variables

**Fig. 3:**
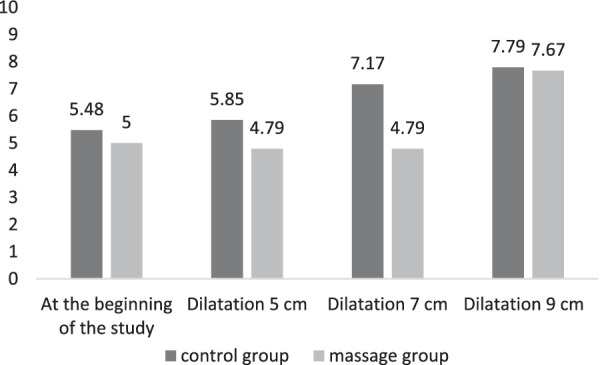
Pain intensity at the beginning of the study and dilatations of 5, 7 and 9 cm

The results of analysis of covariance of pain intensity in different dilatations in the control and massage groups were shown in Table 2. There was no significant difference between two groups at the beginning of the study. There was a significant difference in the intensity of pain between control and massage groups in dilatation of 7 cm while the difference was not significant in dilatation of 5 cm and 9 cm. The means of pain intensity in different dilatations in control and massage group are shown in Fig. 3. As a common rule with the increase of dilatation, the intensity of pain was enhanced. The pain intensity was lower in the massage group compared to control group. The highest decrease of pain intensity was observed at 7 cm dilatation (Fig. 3).

**Table 2:** Mean ± SD pain intensity in different dilatations in control and massage groups

Cervical dilatation	Mean ± SD Study groups		df	f	*p*
	Control	Massage			
At the beginning of the study	5.48 ± 1.55	5.00 ± 1.51	2	0.995	0.376^+^
Dilatation 5 cm	5.58 ± 1.64	4.79 ± 1.30	2	2.58	0.084*
Dilatation 7 cm	7.17 ± 1.37	4.79 ± 1.18	2	30.40	< 0.0001*
Dilatation 9 cm	7.79 ± 0.61	7.67 ± 0.49	2	0.531	0.591*

^+^Independent t-test, *Analysis of covariance (ANCOVA)

**Fig. 4:**
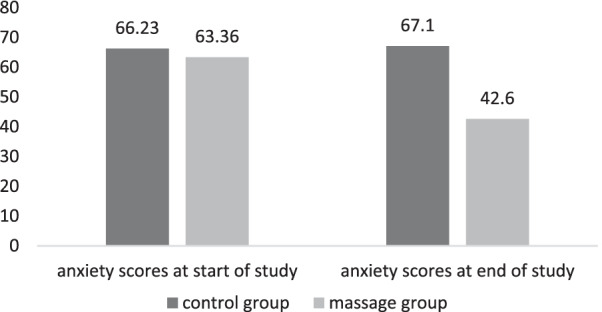
Mean anxiety scores at the start and end of study between groups

The results of analysis of covariance of anxiety in control and massage groups before and after the intervention are presented in Table 3. There was a significant difference in anxiety score between beginning and the end of study (after intervention). The means for anxiety scores in massage and control groups at the beginning and end of the study are shown in Fig. 4. The anxiety score noticeably decreased in massage group anxiety score in the massage group was decreased from 63.36 ± 5.28 (severe anxiety) at the beginning of the study to 42.60 ± 5.83 (moderate anxiety) at the end of the study. In the control group, it increased from 66.33 ± 7.66 to 67.1 ± 5.65.

**Table 3:** Mean ± SD between anxiety scores at the beginning and end of study

Stage of study	Mean ± SD Study groups		df	f	p*
	Control	Massage			
Anxiety scores at the beginning of study	66.33 ± 7.66	63.36 ± 5.28	2	134.36	< 0.0001
Anxiety scores at the end of study	67.10 ± 5.65	42.60 ± 5.83			

*Analysis of covariance (ANCOVA)

The mean duration of labor in the control group was 10.06 ± 1.0 h and in massage group was 9.56 ± 1.6 h, but this difference was not statistically significant (p = 0.17). All of the participants in either the massage group or the control group had not postpartum infection. No side effects and harms were observed in study groups.

## Discussion

In this randomized, -controlled- clinical trial, the appropriate cervical dilatation for applying massage so as reduce the labor pain was obtained in cervical dilatation of 7 cm. According to systematic review, studies showed that massage has effectively reduced the labor pain. In a similar study Gonenc and terzioglu have found that the dual application of massage and acupressure is effective in relieving labor pain [19]. Türkmen and Oran have reported that the mean pain score of heat application group (4.56 ± 0.67) during 4–5 cm of cervical dilation was significantly lower than those in the massage (5.03 ± 1.06) and control groups (5.23 ± 0.72) (p < 0.05). In accord with the results of this study the mean pain scores in heat application group (6.80 ± 0.7) and massage group (7.30 ± 0.8) were significantly lower than that in control group (7.70 ± 0.5) (p < 0.001) at cervical dilatation of 6–7 cm [20].

Erdogan et al. conducted a study to show the effect of back massage on labor pain and maternal satisfaction on 62 samples in Turkey. Among them 31 patients were examined in the massage group and 31 patients in the control group. Massage was performed in three phases of latent, active and transition phases, and pain was assessed three times based on visual criteria. The results showed that lumbar massage had a significant effect on reducing labor pain and maternal satisfaction [9]. In another study, Mohamed et al. assessed the effect of back massage and relaxation techniques on labor in Egypt. Fifty nulliparous pregnant women participated in the study in two study and control groups. In addition, following the application of massage and relaxation techniques, the blood serotonin levels and delivery model have been evaluated. The results of this study showed that lumbar massage is a healthy and inexpensive complementary method to relief labor pain [21]. In Brazil, Gallo et al. conducted a study to detect the effect of massage in reducing the severity of labor pain in a clinical trial. After the massage, pain intensity was measured with a visual criterion of 100 mm. The results showed that the pain intensity was 52 mm in the case group and 72 mm in the control group, similar to our results. The reason for the insignificant difference in means of pain in dilatation of 5 cm can be due to the onset of labor pains and milder pain at this stage. Lack of significant difference in means of pain in 9 cm dilatation can be due to more severe pain and fetal descent and approaching the exit stage of the fetus.

According to the gate control theory, massage stimulates the nerve fibers inhibiting the transmission of painful stimuli arising from the uterine contractions, thereby relieving the pain. Furthermore, application of the massage increases the threshold of the pain tolerance [22]. Manipulation by soft-tissue massage increases local blood circulation and oxygenation in muscle, muscle flexibility, the movement of lymph, and reduces adherent connective tissue. These local effects may influence neural activity at the spinal cord segmental level and could modulate the activities of subcortical nuclei that influence mood and pain perception. Massage may also increase the pain threshold through the release of endorphins and serotonin.

In this study, the mean score of anxiety in participants at the beginning of the study was relatively high. Mothers' anxiety was significantly reduced following the massage, and it seems that physical contact with the mother during childbirth has a potential effect on calming and reducing maternal anxiety. This finding is consistent with the study of Silva et al. reported the high prevalence of anxiety during pregnancy (42.9%) [23]. Barrio and Gasch also pointed out that the intensity of anxiety was four times more in primiparous pregnant women [24]. Chang et al. declared that 87% of participants confirm the effectiveness of massage in decreasing the anxiety [15]. Akköz and Karaduman also pointed out that sacral massage applied during labor reduced the women's anxiety [25], the results that were in accordance with the present study.

In this survey, there was no significant difference in mean duration of labor between the control and massage groups. Gonenc and Terzioglu declared that there were no significant differences between the acupressure and massage groups and the control group in terms of delivery time [19] that is similar to the result of this study. Sulistiningsih et al. (2022) reported a significant reduction in duration of labor, in contrary to the current study [12].

A literature review showed that numerous studies have been performed in this field, but far too little attention has been paid for studying appropriate cervical dilatation for massage to reduce labor pain. Moreover, the application of massage in the delivery rooms was rare. The prevalence of CS is much higher than what WHO has recommended. It seems to be essential to decrease such a phenomenon by eliminating the mothers’ fear of vaginal delivery. Since the most common cause of cesarean section is the history of previous cesarean (42.25%) [1], the safely prevention of primary cesarean delivery is important, and reducing the labor pain with massage is in regard. Strengths of study was controlled received massage and the uniformity of the intervention, since massage, cervix examination and measurements were performed by the same researcher. In the control group, the routine maternity ward care was performed except massage. The too long sampling time and also an impossibility of blinding were two limitations of the current study.

## Conclusion

The most effective massage time to reduce labor pain was observed in cervical dilatation of 7 cm. In order to support vaginal delivery and promote maternal health, the massage at cervical dilatation of 7 cm is recommended as a routine care especially in nulliparous women. The application of massage can also reduce the maternal anxiety.

## Data Availability

The datasets generated during and analyzed during the current study are available from the corresponding author.

## Abbreviations

CS: Cesarean section
BMI: Body mass index
LDR: Labor, delivery, and recovery
ANCOA: Analysis of covariance
